# Supplementary material for: A UMLS-based spell checker for natural language processing in vaccine safety
Source: BMC Med Inform Decis Mak. 2007 Feb 12;7:3. doi: 10.1186/1472-6947-7-3 (PMC1805499; doi:10.1186/1472-6947-7-3)
Supplement: Additional file 13 — RAP application directory. Sets up directory for RAP (RDF application for PHP) source code [file 1472-6947-7-3-S13.gz › rap/doc/phpdoc/__filesource/fsource_resModel__resModelResResource.php.html]

xml version="1.0" encoding="iso-8859-1"?


File Source for ResResource.php


# Source for file ResResource.php

Documentation is available at ResResource.php

```
1. <?PHP
2. /**
3. * ----------------------------------------------------------------------------------
4. * Class: ResResource
5. * ----------------------------------------------------------------------------------
6. * @package resModel
7. ***/

9. /**
10. * An RDF Resource.
11. * Resource instances, when created, are associated with a specific model. They support a
12. * range of methods, such as getProperty() and addProperty() which will access or modify
13. * that model. This enables the programmer to write code in a compact and easy style.
14. *
15. * <BR><BR>History:<UL>
16. * <LI>10-01-2004                : First version of this class.</LI>
17. *
18. * @version  V0.9.1
19. * @author Daniel Westphal <mail at d-westphal dot de>
20. *
21. *
22. * @package resModel
23. * @access    public
24. ***/
25. class ResResource extends Resource
26. {
27. /**
28. * Holds a reference to the associated model
29. * @var        ResModel
30. * @access    private
31. */
32. var $model;

34. /**
35. * Is true, if this resource is an anonymous node.
36. * @var        boolean
37. * @access    private
38. */
39. var $isAnon;


42. /**
43. * Constructor
44. * You can supply a uri
45. *
46. * @param string $uri
47. * @access    public
48. */
49. function ResResource($uri)
50. {
51. parent::Resource($uri);
52. $this->isAnon = ($uri === null);
53. }

55. /**
56. * Sets the reference to the assocoated model.
57. *
58. * @param    object Model    $model
59. * @access public
60. */
61. function setAssociatedModel(& $model)
62. {
63. $this->model=& $model;
64. if ($this->isAnon)
65. $this->uri=$this->model->getUniqueResourceURI(BNODE_PREFIX);
66. }

68. /**
69. * Get the reference to the assocoated model.
70. *
71. * @return    object Model    $model
72. * @access public
73. */
74. function getAssociatedModel()
75. {
76. return $this->model;
77. }

79. /**
80. * Sets the URI of this resource
81. *
82. * @param    string $uri
83. * @access public
84. */
85. function setURI($uri)
86. {
87. $this->uri = $uri;
88. }

90. /**
91. * Add a property to this resource.
92. * A statement with this resource as the subject, p as the predicate and o
93. * as the object is added to the model associated with this resource.
94. *
95. * @param    ResResource                $property
96. * @param    ResResource/ResLiteral    $object
97. * @return    object ResResource
98. * @access    public
99. */
100. function addProperty($property,$object)
101. {
102. $this->model->add(new Statement($this,$property,$object));

104. return $this;
105. }

107. /**
108. * List all the values with the property p as statements in an array.
109. *
110. * @param    ResResource        $property
111. * @return    ResIterator
112. * @access    public
113. */
114. function listProperties($property = null)
115. {
116. return $this->model->find($this,$property,null);
117. }

119. /**
120. * Answer some statement (this, p, O) in the associated model.
121. * If there are several such statements, any one of them may be returned.
122. * If no such statements exist, null is returned.
123. *
124. * @param    ResResource                $property
125. * @return    object ResResource
126. * @access    public
127. */
128. function getProperty($property)
129. {
130. return $this->model->getProperty($this,$property);
131. }

133. /**
134. * Determine whether this resource has any values for a given property.
135. *
136. * @param    ResResource        $property
137. * @param    ResResource        $value
138. * @return    object ResResource
139. * @access    public
140. */
141. function hasProperty($property, $value = null)
142. {
143. $ret= $this->model->findFirstMatchingStatement($this,$property,$value);

145. return ($ret!==null);
146. }

148. /**
149. * Determine whether this resource is an anonymous resource
150. *
151. * @return    boolean
152. * @access    public
153. */
154. function getIsAnon()
155. {
156. return $this->isAnon;
157. }

159. /**
160. * Set whether this resource is an anonymous resource
161. *
162. * @param    boolean
163. * @access    public
164. */
165. function setIsAnon($isAnon)
166. {
167. $this->isAnon=$isAnon;
168. }

170. /**
171. * Checks if the resource equals another resource.
172. * Two resources are equal, if they have the same URI
173. *
174. * @access    public
175. * @param    object    resource $that
176. * @return    boolean
177. */
178. function equals ($that)
179. {
180. if (is_a($that,'ResLiteral'))
181. return $that->equals($this);

183. return ($that!==null && ($this->getURI() == $that->getURI()));
184. }

186. /**
187. * Delete all the statements with predicate p for this resource from
188. * its associated model.
189. *
190. * @access    public
191. * @param    object    resource $property
192. * @return    object ResResource
193. */
194. function removeAll($property = null)
195. {
196. foreach ($this->model->find($this,$property,null) as $statement)
197. {
198. $this->model->remove($statement);
199. }
200. return $this;
201. }

203. /**
204. * Delete all the properties for this resource from the associated model.
205. *
206. * @access    public
207. * @return    object ResResource
208. */
209. function removeProperties()
210. {
211. $this->removeAll();
212. return $this;
213. }
214. }
215. ?>
```

Documentation generated on Thu, 7 Jul 2005 13:43:48 +0200 by phpDocumentor 1.3.0RC3
